# Accuracy of Tooth Segmentation in the Digital Kesling Setup of Two Different Software Programs: A Retrospective Study

**DOI:** 10.7759/cureus.70306

**Published:** 2024-09-27

**Authors:** Rebekah Raju, Prasanna Aravind TR

**Affiliations:** 1 Department of Orthodontics and Dentofacial Orthopaedics, Saveetha Dental College and Hospitals, Saveetha Institute of Medical and Technical Sciences, Saveetha University, Chennai, IND

**Keywords:** accuracy, digital planning, intraoral scans, kesling setup, tooth segmentation

## Abstract

Introduction

Precise virtual setup creation and orthodontic appliance fabrication depend on accurate teeth segmentation from intraoral scans. This accuracy is also fundamental for successful orthodontic treatment, as it ensures correct diagnosis and optimal treatment planning. A number of software packages that facilitate the building of virtual setups have been made available in recent years. The performance of these software packages on automatic tooth segmentation has not been widely studied. Hence, the aim of this study was to evaluate the accuracy of automated teeth segmentation in the digital Kesling setup of Ortho Studio (Maestro 3D Dental Studio, Bordeaux, France) and OrthoAnalyzer (3Shape, Copenhagen, Denmark) software systems.

Materials and methods

All the scans were taken from the same intraoral scanner (Runyes 3D intraoral scanner; Runyes Medical, Ningbo, China). The scans were stored and imported as stereolithography (STL) files into the Maestro Ortho Studio and 3Shape OrthoAnalyzer software systems. Subsequently, the digital photos underwent alignment in both software applications, an essential stage in each respective workflow prior to any further processing. The digitized images were automatically segmented in Maestro and 3Shape software by a single researcher. For each software interface, the accuracy of teeth segmentation was assessed. An independent t-test, with a significance level set at p < 0.05, was used to evaluate the statistical significance between the two software segmentations.

Results

The total number of teeth segmented by both software programs utilizing the 12 intraoral scans was 336 for both groups. Successful identification of the tooth segments was 98.21% (n = 330) for 3Shape software and 98.8% (n = 332) for Maestro software. There was no significant difference in the accuracy of determining the tooth segmentations between anterior and posterior teeth, respectively, between both groups, with a p-value of 0.523.

Conclusion

There were no statistically significant differences between the two software programs, and both demonstrated high success rates for auto-tooth segmentation. Although both programs had excellent success rates, Maestro 3D performed more accurately than 3Shape OrthoAnalyzer.

## Introduction

In the rapidly evolving field of orthodontics, digital imaging has revolutionized traditional practices by providing a meticulous anatomical perspective essential for diagnosis and optimized treatment strategies [1]. The advent of technologies such as digital record-keeping, digital cast models, occlusograms, and intraoral scanners has propelled orthodontic practice to unprecedented heights, integrating sophisticated 3D computer models that facilitate indirect bracket placement for customized orthodontic devices [2]. This shift from conventional methods to digitization - encompassing innovations like clear aligners, computer-aided design/computer-aided manufacturing (CAD/CAM), 3D printing, and 3D cephalometry - has dramatically enhanced diagnostic precision, streamlined treatment planning, and strengthened patient compliance by reducing both clinician effort and chair-side time [3-5]. A thorough examination of the patient's oral health is the initial phase in the aligner planning process [6]. Following this, digital scanning is used to produce accurate 3D models of the teeth. The gradual movement of teeth is then simulated using specialist software to create a personalized treatment plan. After the patient's delivery of the aligners and their use, follow-up evaluations guarantee that the treatment is proceeding according to plan, with any required modifications. Finally, a review is required to confirm the ideal alignment, and retainers are provided to maintain the teeth in the new position. Precise virtual setup creation and orthodontic appliance fabrication depend on accurate teeth segmentation from intraoral scans. This accuracy is also fundamental for successful orthodontic treatment, as it ensures correct diagnosis and optimal treatment planning [7,8]. To enhance the segmentation of 3D dental models, various deep learning techniques have been developed, such as the region-based and feature curve-based approaches [9]. Region-based methods rely on the similarity of surrounding regions to distinguish different parts of the mesh. In contrast, feature curve-based methods offer more refined segmentation. Hence, the latter is more prominently adapted for tooth segmentation due to its ability to adapt to the intricate contours of dental anatomy, providing more accurate and effective segmentation results [10]. A number of software packages that facilitate the building of virtual setups have been made available in recent years. Some of the often-used systems are OrthoAnalyzer (3Shape, Copenhagen, Denmark), Ortho Studio (Maestro 3D Dental Studio, Bordeaux, France), OrthoCAD (Align Technology, San Jose, CA, USA; AGE Solutions, Pontedera, Italy), and uDesign (uLab Systems, Inc., Memphis, TN, USA) [11].

High precision in segmenting orthodontic models enables better monitoring of tooth movement and treatment progression, facilitating timely adjustments to orthodontic plans and minimizing errors. However, the performance of the aforementioned software on automatic tooth segmentation has not been widely studied [12]. Hence, the aim of this study was to evaluate the accuracy of automated teeth segmentation in the digital Kesling setup of Ortho Studio and OrthoAnalyzer software systems.

## Materials and methods

Study setup

This retrospective study was conducted at the Department of Orthodontics, Saveetha Dental College and Hospitals, Chennai, India. The pretreatment records of the patients were assessed for eligibility based on the selection criteria. The sample size was determined based on a previous study by Yacout et al., which found that the sample size was 12 [13]. Patients' informed consent, allowing for the use of their anonymized records for research purposes, was obtained before the start of the study. The study was approved by the Institutional Review Board with the ethical number: IHEC/SDC/RS/ORTHO-2101/24/173.

Selection criteria

The inclusion criteria included the intraoral scans of patients aged 18 years and older with permanent dentition, presenting with moderate to severe crowding in the anterior teeth region. The digital dental models were required to be free from any defects in the teeth and/or gingiva. Digital dental models with crowns, multiunit bridges, partially erupted teeth, or missing or supernumerary teeth were excluded based on the findings in the OPG of the participants.

Intraoral scan records

All the scans were taken from the same intraoral scanner (Runyes 3D intraoral scanner; Runyes Medical, Ningbo, China). The scans were saved and imported in stereolithography (STL) file format into the Maestro Ortho Studio and 3Shape OrthoAnalyzer software systems. The digital images were then aligned in both software programs, which was a prerequisite step in each program's workflow before additional processing. The digitized images were automatically segmented in the Maestro and 3Shape software by a single researcher. For each software interface, the accuracy of teeth segmentation was assessed.

Automatic tooth segmentation evaluation

**Figure 1:**
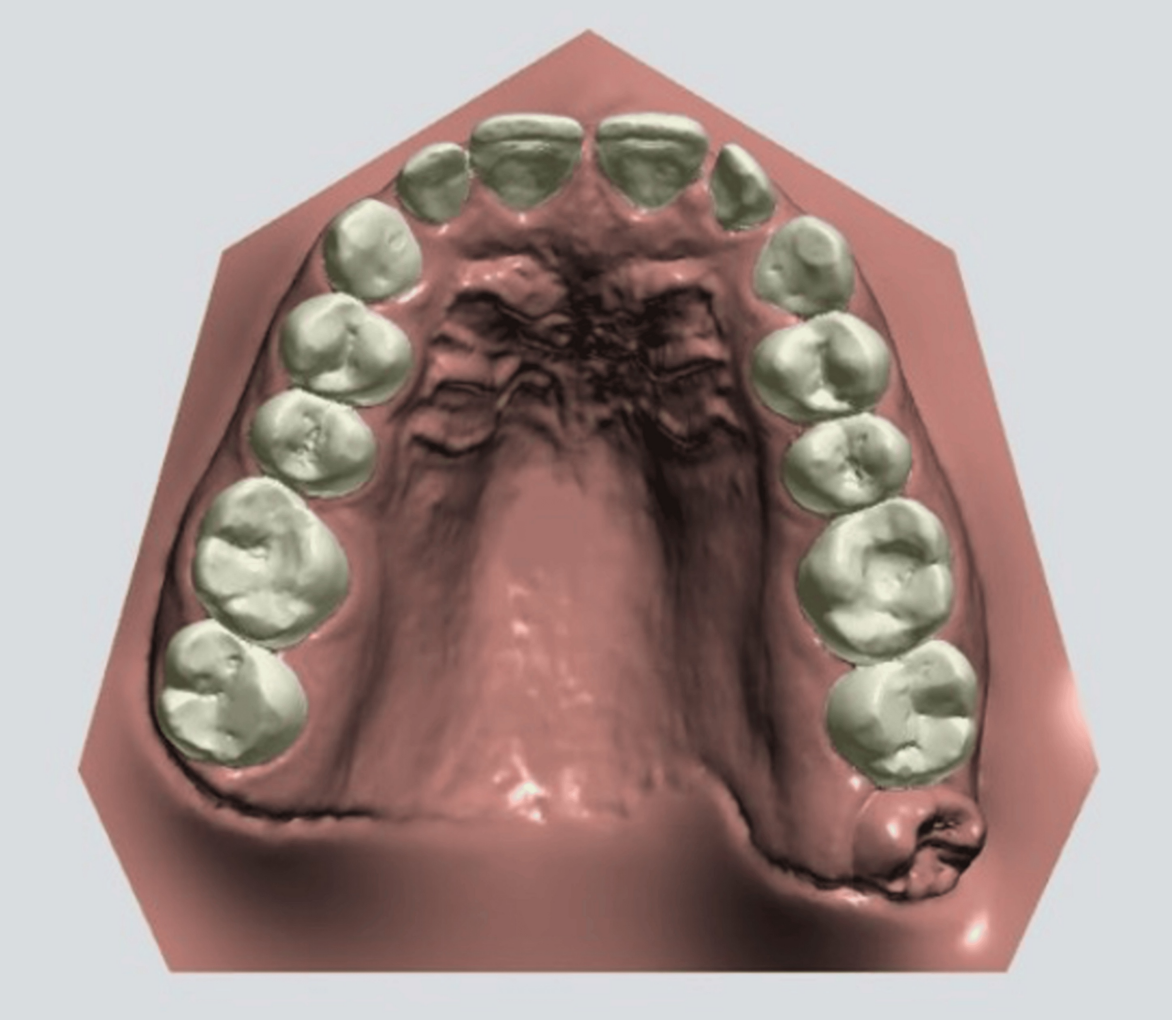
Automatic teeth segmentation using 3Shape software

**Figure 2:**
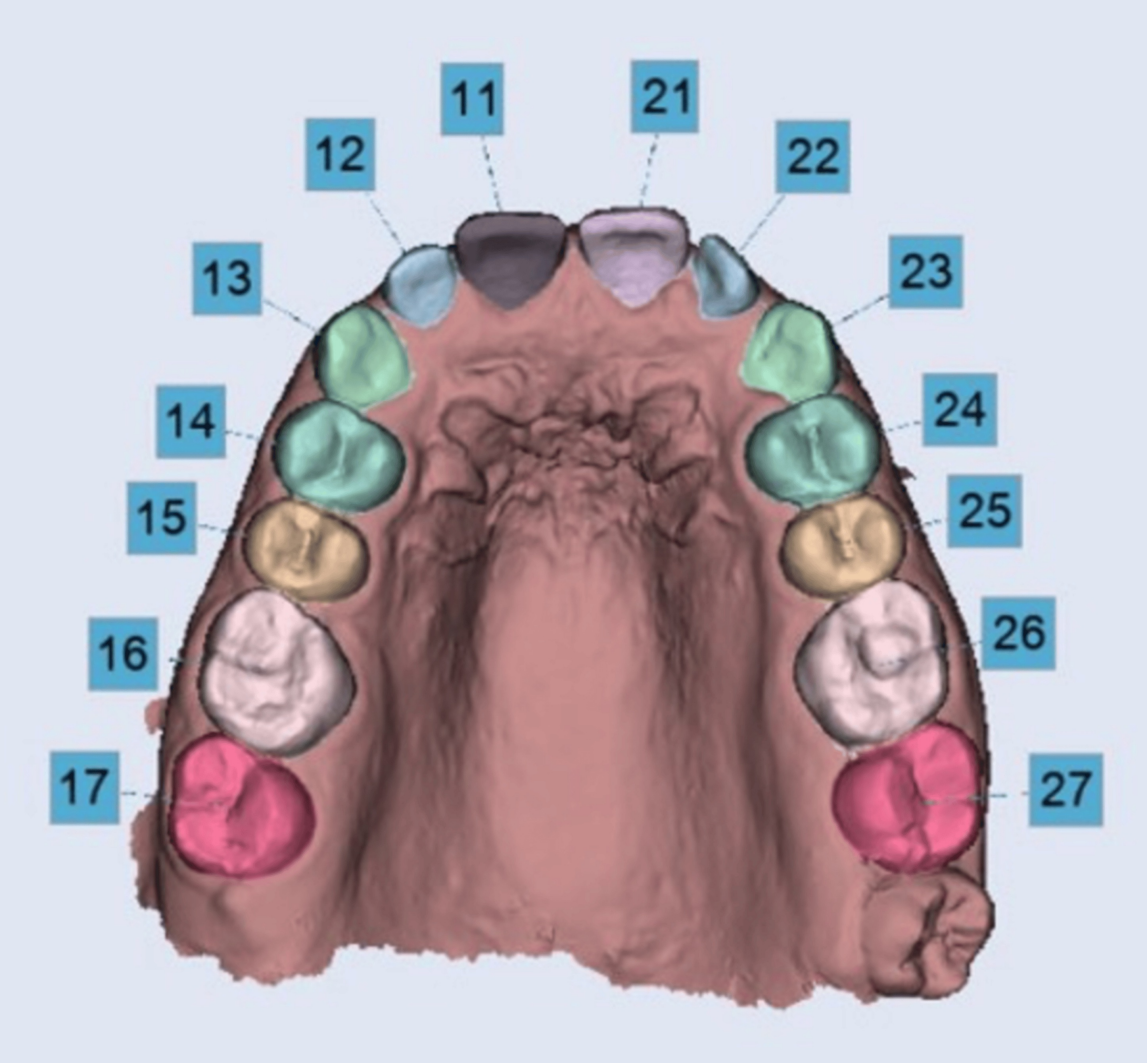
Automatic teeth segmentation using Maestro software

On both software programs, automatic segmentation was performed (Figures 1-2). The success rate of each program's automated segmentation is based on whether the entire tooth surface (especially the mesial and distal surfaces) is covered. It also depended on the amount of excess surface covered and how the long axis of the tooth was generated. Based on the above-mentioned criteria, success or failure was determined. The quality of segmentation was assessed for the teeth that were identified. If the segmented scan covered the entire tooth surface up to the gingival margin, the segmentation was deemed adequate. Segments with errors were categorized based on the surface implicated (lingual, vestibular, or occlusal) and whether the defect was a surplus (vestibular and/or lingual) or a deficit (occlusal, vestibular, or lingual).

Statistical analysis

The statistical analysis was performed using IBM SPSS Statistics for Windows, Version 23 (Released 2015; IBM Corp., Armonk, NY, USA). The values of the mean and standard deviation were computed. The percentage of successful automatic segmentation of teeth in Maestro and 3Shape software was calculated individually. An independent t-test, with a significance level set at p < 0.05, was used to evaluate the statistical significance between the two software segmentations.

## Results

**Figure 3:**
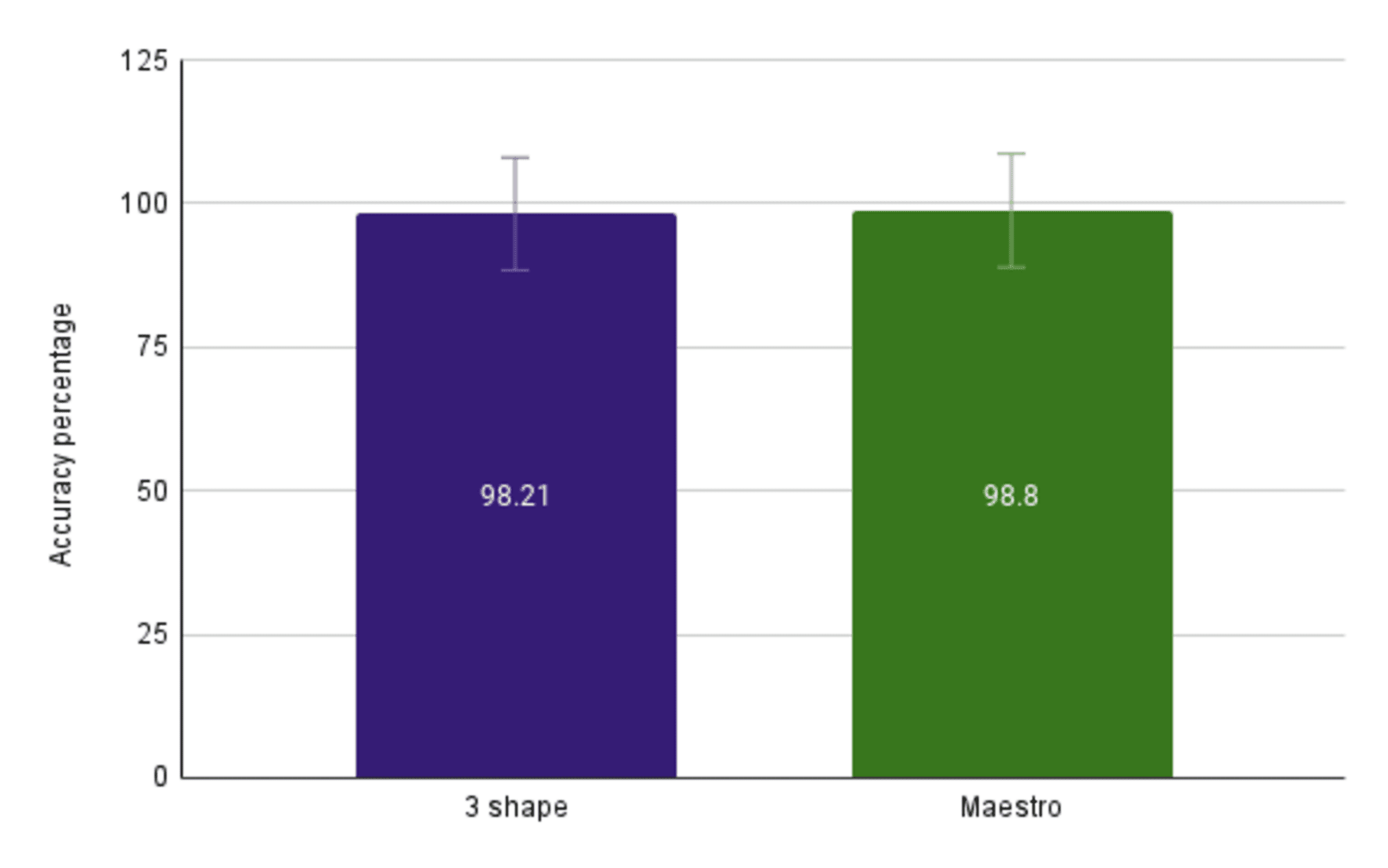
Percentage of successful automatic teeth segmentation 3Shape: 3Shape OrthoAnalyzer software; Maestro: Maestro Ortho Studio software

In this study, the total number of teeth segmented by both software programs, utilizing the intraoral scans of all the included patients, was 336 for both groups. The descriptive statistics have been tabulated in Table 1. Successful identification of the tooth segments was 98.21% (n = 330) for 3Shape software and 98.8% (n = 332) for Maestro software (Figure 3). This indicated that Maestro performed marginally superior in terms of tooth segmentation accuracy. With a p-value of 0.523, the results indicated that there was no statistically significant difference in accuracy between these two groups of teeth for either software, suggesting that both systems functioned consistently across various tooth types (Table 2). Thus, while Maestro had a slight edge in overall accuracy, both software programs demonstrated comparable performance in automatic tooth segmentation.

**Table 1:** Descriptive statistics Mean of the total number of accurate teeth segmentations N = number of intraoral scan samples

Digital software setup	Mean	Standard deviation	N
3Shape	27.5	0.67	12
Maestro	27.66	0.49	12

**Table 2:** Independent t-test Level of significance (p < 0.05)

Parameter	df	t	Significance (p-value)
Accuracy of teeth segmentation	21	0.64	0.52

## Discussion

Research on automatic tooth segmentation in intraoral scanning gained momentum due to advancements in dental imaging technology and the need for efficient dental diagnostics and treatments [13,14]. The present study evaluated the accuracy of tooth segmentation in the digital Kesling setup of 3Shape OrthoAnalyzer software and Maestro digital software. The accuracy ranged from 98.21% to 98.8%. Based on the results of the current study, the accuracy of Maestro digital software was found to be clinically higher when compared to the 3Shape software. These results were similar to the findings obtained by Barreto et al. [15], in which the gingival margin was not clearly discernible using OrthoAnalyzer software. Regarding the identification of teeth, there was no statistically significant difference seen between the two software packages. This was consistent with the high success rates in tooth segmentation, as successful segmentation commonly occurred in the aforementioned software. Due to variations in the methodologies used, it was difficult to compare the success rate of tooth segmentation in the current study with data from earlier publications. An automatic segmentation success percentage of 97.26% was obtained by Im et al. [9]. However, in their investigation, each instance of a segmentation error - excessive or deficient - on any tooth surface was regarded as a segmentation failure. Furthermore, the failures were not categorized based on the various tooth types.

A standardized accuracy evaluation technique was proposed by Vitai et al. [16], who also recommended using laboratory scanners to provide reference data. Wang et al. [17] provided an efficient and simplified tool for tooth segmentation using the convolutional neural network (CNN) model, which was validated for further integration into clinical practice. Xu et al. [18] stated that the final step, segmentation, involved the deployment of deep learning models, notably convolutional neural networks, to classify and separate each tooth based on the refined features. This method ensured the generation of highly accurate digital models, which significantly enhanced the precision of dental diagnostics and treatment planning.

Based on the literature evidence, several benefits associated with automatic segmentation have been identified. It significantly increases operational efficiency by processing and analyzing dental scans more quickly. Borbély [19] stated that this technique easily merges with digital workflows, such as 3D printing and CAD/CAM systems, making it easier to create extremely precise orthodontic appliances and restorations. Lee et al. [20] confirmed improved segmentation accuracy, which could be utilized much more effectively, allowing for greater in-depth studies of tooth alignment and location for both treatment planning and diagnosis. On the contrary, the accuracy of automatic tooth segmentation is also hindered by several intricate challenges. The complexity of gingival margins presents a significant hurdle, as their subtle and highly variable nature often leads to inaccuracies in precise delineation [16]. Additionally, segmentation becomes problematic in interproximal areas, especially in cases of dental crowding or overlapping teeth, where artificial intelligence (AI) algorithms struggle to differentiate individual teeth accurately [17,21]. The quality and variability of data could further complicate matters, with factors such as patient movement or inherent scanner limitations affecting the clarity and consistency of intraoral scans [9]. Training and validation of AI models demand extensive and diverse datasets to ensure effectiveness across varied patient anatomies, making validation crucial for achieving reliable performance in diverse clinical contexts [19].

Recent investigations into automated tooth segmentation using advanced deep-learning techniques have highlighted substantial enhancements in the analysis of intraoral scans [20]. Im et al. [9] performed a comparative study among various methods, including landmark-based approaches (OrthoAnalyzer), tooth designation strategies (Autoalign), and deep learning algorithms, discovering that the latter achieved an impressive success rate of 97.26%. Kim et al. [22] introduced a novel automated tool employing generative adversarial networks to reconstruct missing interdental areas from intraoral scans, demonstrating superior performance relative to conventional plaster cast techniques.

Based on the available evidence from the literature, it can be confirmed that automatic teeth segmentation is very relevant from a clinical perspective, as it enhances bracket placement accuracy and treatment planning. It facilitates efficient workflow optimization, lowers human error, and allows for effective monitoring of tooth movement. Thus, the present study indicated that digital scanning and tooth segmentation techniques were reliable and accurate, with better identification of tooth segments.

Limitations

Several limitations affect the accuracy and reliability of intraoral scans for dental analysis. Variations in tooth morphology, such as dental wear or abnormalities throughout development, make segmentation and modeling even more difficult and could lead to inaccurate digital representations. The user-friendliness and cost of the software were not taken into consideration in the study. Furthermore, intraoral scan aberrations, such as noise and distortions caused by the patient's movement, the constraints of the scanner, or reflective surfaces, could mask tooth morphology and reduce the quality of the images taken.

## Conclusions

According to the results of this investigation, there were no statistically significant differences between the two software programs, and both demonstrated high success rates for auto-tooth segmentation. Although both programs had excellent success rates, Maestro 3D performed more accurately than 3Shape OrthoAnalyzer. Additional research is necessary to verify the effectiveness of these software programs and ensure precision.
